# Surface and subsurface dispersal of radioactive materials from Fukushima by subpolar gyre and intermediate waters in the North Pacific

**DOI:** 10.1038/s41598-024-55328-7

**Published:** 2024-03-01

**Authors:** Seung-Tae Lee, Yang-Ki Cho, Jihun Jung, Seunghwa Chae

**Affiliations:** 1 Department of Ocean Sciences, University of California, 1156 High Street, Santa Cruz, CA USA; 2 School of Earth and Environmental Sciences, Research Institute of Oceanography, Seoul National University, Seoul, Korea; 3 College of Earth, Ocean and Atmospheric Sciences, Oregon State University, Corvallis, OR USA

**Keywords:** Environmental sciences, Ocean sciences

## Abstract

Radioactive materials were released into the ocean following the Fukushima Daiichi Nuclear Power Plant accident in 2011. Six years after the accident, the radioactive material concentration was markedly increased in the Okhotsk Intermediate Water (OIW) of the Sea of Okhotsk. This material may have been subjected to southward subsurface dispersal by the North Pacific Intermediate Water (NPIW), which originates from the OIW. The spatiotemporal limitations of available methods have made it challenging to track the dispersal paths of radioactive materials in the North Pacific Subpolar region. Here, we performed a tracer experiment using a three-dimensional numerical model to determine the path of ^137^Cs from Fukushima to the Sea of Okhotsk via surface subpolar gyre currents and subsurface dispersion by OIW and NPIW. The results showed that the ^137^Cs concentration in the Sea of Okhotsk increased via the surface current and moved progressively southward via OIW six years after the accident and eastward via OIW and NPIW nine years after the accident, indicating that ^137^Cs transported by NPIW entered the subtropical region. Based on experiments, this temporal change was mainly caused by ocean currents. Thus, subsurface recirculation of radioactive material via the OIW and NPIW should be considered based on the predicted path and travel time of additional materials released from the power plant.

## Introduction

**Figure 1:**
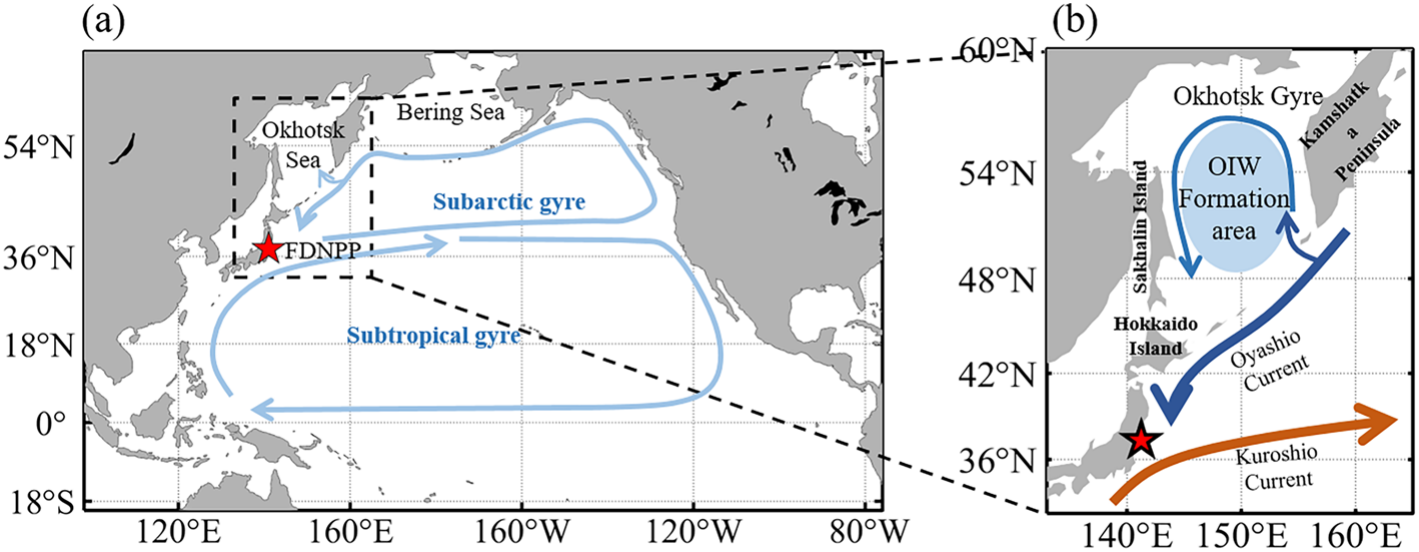
(**a**) Model domain and surface current system in the North Pacific region. Red star represents the location of the Fukushima Daiichi Nuclear Power Plant (FDNPP). (**b**) Major surface currents in the Okhotsk Sea and formation area of the Okhotsk Intermediate Water (OIW). Figures were generated by S-TLee using MATLAB R2020a (http://www.mathworks.com).

Large quantities of radioactive material were released following the Fukushima Daiichi Nuclear Power Plant (FDNPP) accident on March 11, 2011. This event resulted in an estimated addition of approximately 3.5 PBq to the ocean ^137^C inventory^1^. Released radioactive materials may be transported primarily by currents. The North Pacific has two large current systems: a subtropical gyre and subarctic gyre (Fig. 1a). The Oyashio Current flows to the Kuroshio Extension in the western subarctic North Pacific facing the Sea of Okhotsk. Part of the Oyashio Current enters the Sea of Okhotsk, creating a counterclockwise Okhotsk gyre (Fig. 1b).

Numerous observational studies have been performed to monitor the paths of radioactive materials following the accident, which showed that surface radioactive material mainly spreads eastward through the Kuroshio extension^2–4^. Notably, high concentrations of radioactive materials have also been observed in the subsurface of the southern area of the Kuroshio Extension^5–7^; these high subsurface concentrations can be dispersed by mode waters^8,9^. Most studies focused on surface dispersion by the subtropical gyre and subsurface dispersion by mode water in the North Pacific.

Routine observations in the Bering Sea and Sea of Okhotsk revealed a significant increase in the concentration of radioactive Cs in the subsurface six years after the FDNPP accident^10,11^. These observations suggested that FDNPP-derived radionuclides were transported to the Arctic Ocean via the Bering Sea by the subpolar gyre^10^. The elevated concentration of radioactive Cs on the southern coast of the Sea of Okhotsk persisted until 2021, with the maximum concentration observed in the subsurface layer^12^. Additionally, the concentration of ^134^Cs in the southwest region of the Sea of Okhotsk increased, including in the intermediate cold water^13^.

Cold, fresh Okhotsk Intermediate Water (OIW) in the Sea of Okhotsk contributes to formation of the North Pacific Intermediate Water (NPIW)^14–16^. The NPIW plays an important role in the transport of low-salinity water to low latitudes^17^. Furthermore, intermediate waters from the Sea of Okhotsk absorb large amounts of anthropogenic CO_2_ from the atmosphere into the ocean. The OIW and NPIW contain extremely high Fe concentrations^18^. Thus, the OIW and NPIW constitute important water masses for both oceanic and ecological environments in the North Pacific.

The volume transport of the OIW contributes to formation of the NPIW, which has been estimated to be 2.8 Sv^14^. Half of the OIW moves south along the coastline, entering the subtropical gyre beyond the Sub-Arctic Front (SAF), whereas the other half moves offshore along the SAF. In contrast, another possible path was reported via which the inflow path of the NPIW is more apparent offshore than in coastal areas^16^. Therefore, a high concentration of radioactive material subducted into the Sea of Okhotsk may have moved southward to the SAF via the OIW and entered the subtropical gyre by means of the NPIW.

However, observations have not fully revealed the path of radioactive materials in the subpolar region because of the spatiotemporal limitations of available methods. Particularly, studies are needed to determined why the maximum concentration of radioactive materials was observed in the Okhotsk Sea at six years following the accident. Therefore, the subduction and subsurface dispersion of radioactive materials by the NPIW in the subpolar region should be investigated. In this study, we performed three-dimensional numerical modeling and tracer experiments to predict the surface path of radioactive materials and their travel time from Fukushima to the Sea of Okhotsk by subpolar gyre, along with their subsurface dispersion by the OIW and NPIW.

## Results and discussion

### Increase of radioactive material concentration in the Sea of Okhotsk via subpolar gyre

**Figure 2:**
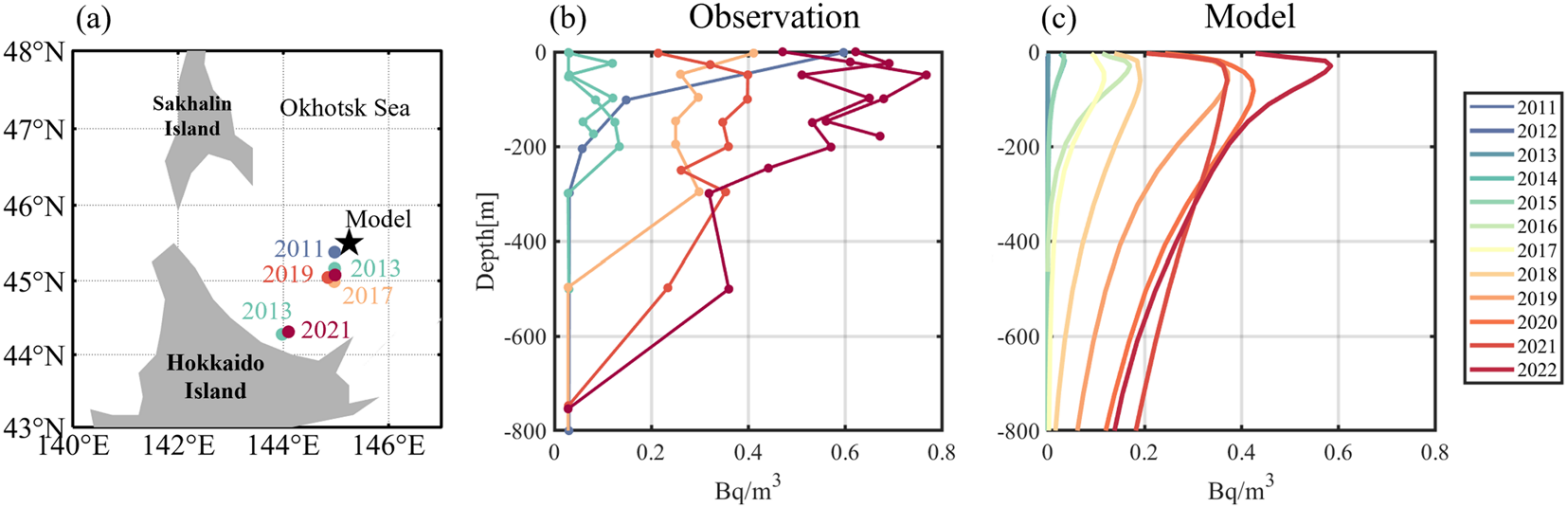
Comparison of observations and model simulation data in the southern area of the Sea of Okhotsk. (**a**) Observation stations utilized by Inoue et al.^13^ and the selected model point in the current study. (**b**) ^134^Cs concentrations decay-corrected to the date of the FDNPP accident in July, from Inoue et al.^13^. (**c**) Simulated ^134^Cs concentrations in July without the half-life decay of ^134^Cs. Figures were generated by S-TLee using MATLAB R2020a (http://www.mathworks.com).

Routine observations of radioactive Cs have been conducted in the Sea of Okhotsk, Japan. Inoue et al. (2022) observed radioactive Cs profiles in the southern area of the Sea of Okhotsk in 2011, 2013, 2017, 2019, and 2021 and estimated the ^134^Cs concentration by decay-correcting each observed ^134^Cs to the date of the FDNPP accident (Fig. 2a). Figure 2a depicts the observation stations from the previous study^11^ and the model grid used to compare the results. Figure 2b shows the decay-corrected ^134^Cs concentrations on the date of the FDNPP accident. Figure 2c shows the simulated concentration profile of ^134^Cs without half-life decay to maintain consistency with the real-world observations. The ^134^Cs concentration after 2017 was approximately seven times higher than that in 2013 (Fig. 2b). Our model results also simulated a marked increase in the ^134^Cs concentration in the Sea of Okhotsk over time, corresponding to real-world observations. Compared with direct ^134^Cs observations, our method has the advantage of tracking only the amount leaked to Fukushima without considering background radioactive material, which has a half-life of approximately 2.1 years. The model results showed that the concentration of ^134^Cs began increasing in 2016 and continued to increase until 2022, whereas the observed concentration of ^134^Cs began increasing in 2017. Considering that no real-world observations were made in 2016, the model accurately simulated the temporal variation in the observed concentration.

The model results accurately simulated the concentration increases at both the surface and subsurface. The model concentration of ^134^Cs increased from 2018 at depths greater than 400 m, corresponding to the increase in the observed concentration of ^134^Cs from 2019 at the same depth. No observations were made in 2018. The maximum concentration of ^134^Cs appeared at a depth of approximately 50–100 m in both the observational and model results after 2016. The depth of the maximum concentration increased over time, suggesting the subduction of radioactive materials from the surface to the subsurface. Nevertheless, despite a similar change in the vertical profile over time, the absolute concentration of the model result was slightly lower than that of the observation. This difference may have resulted from the exclusion of atmospheric deposition of radioactive materials in our model experiment. Our model results suggest that the increase in the observed concentration of Cs in the Okhotsk Sea six years following the Fukushima accident resulted from surface circulation.

**Figure 3:**
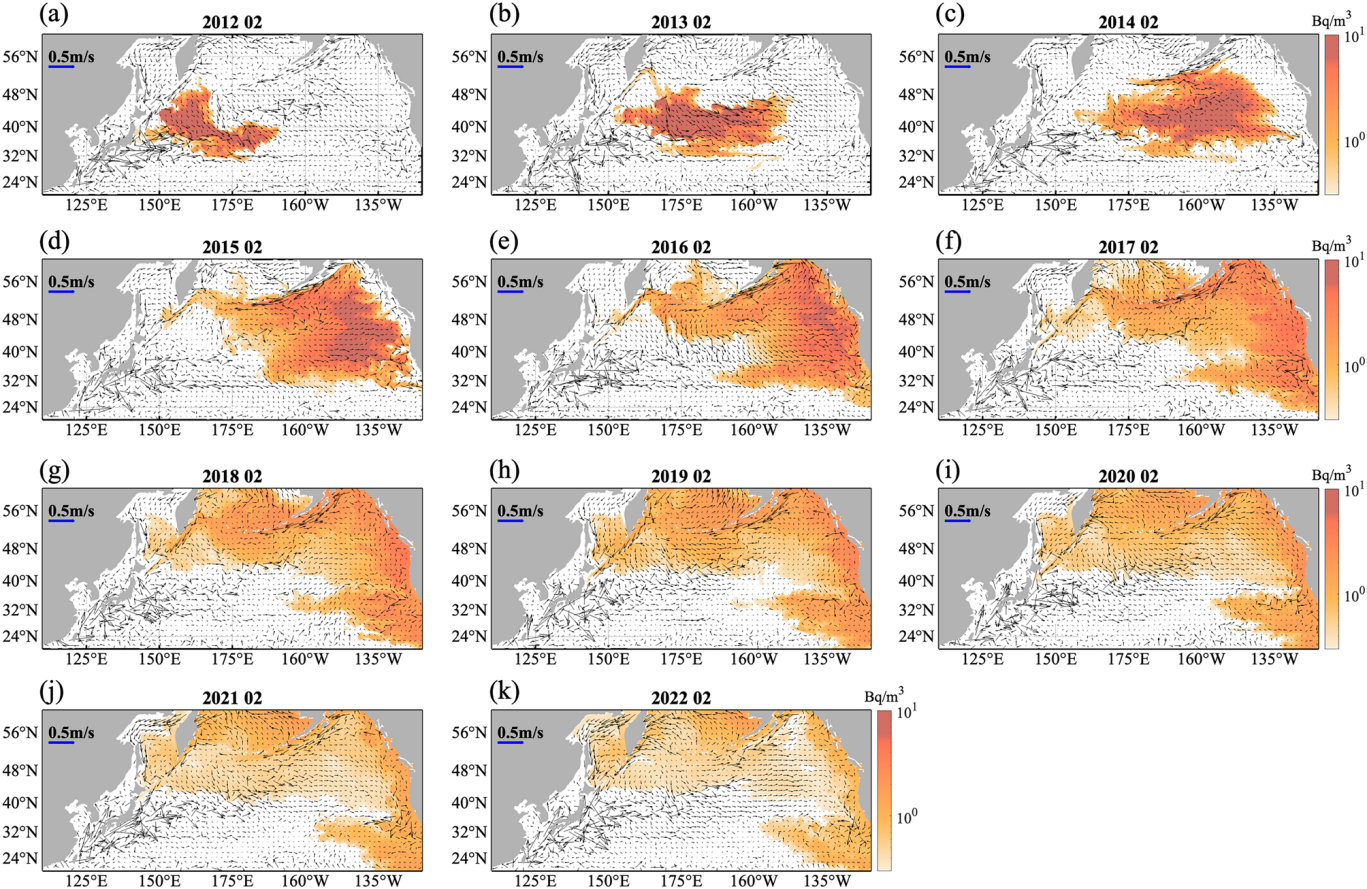
Monthly mean surface distribution of ^137^Cs concentration and surface current every February from (**a**–**k**) 2012 to 2022, based on the model results. Figures were generated by S-TLee using MATLAB R2020a (http://www.mathworks.com).

We simulated the concentration of ^137^Cs, which has a longer half-life than ^134^Cs. The ^134^Cs/^137^Cs activity ratio released from the FDNPP was nearly 1; a similar overall amount of both radioactive Cs was released^19,20^. Figure 3 illustrates the concentration of ^137^Cs in the surface layer from February 2012 to February 2022, revealing that ^137^Cs moved eastward along the Kuroshio extension, and then along the clockwise subtropical gyre and counterclockwise subarctic gyre. ^137^Cs reached the northern boundary of the subarctic gyre in 2014 (Fig. 3c) and the inlets of the Bering and Okhotsk Seas in 2015 (Fig. 3d). ^137^Cs appeared widely in the Okhotsk Sea beginning in 2017, after which its concentration increased continuously (Fig. 3g–k).

The concentration of ^137^Cs was increased in the subsurface of the Bering Sea six years following the accident^10^. Our model also simulated this increase over time, particularly in the density layer of the OIW.

### Subsurface spreading of radioactive materials by OIW and NPIW from the Sea of Okhotsk

**Figure 4:**
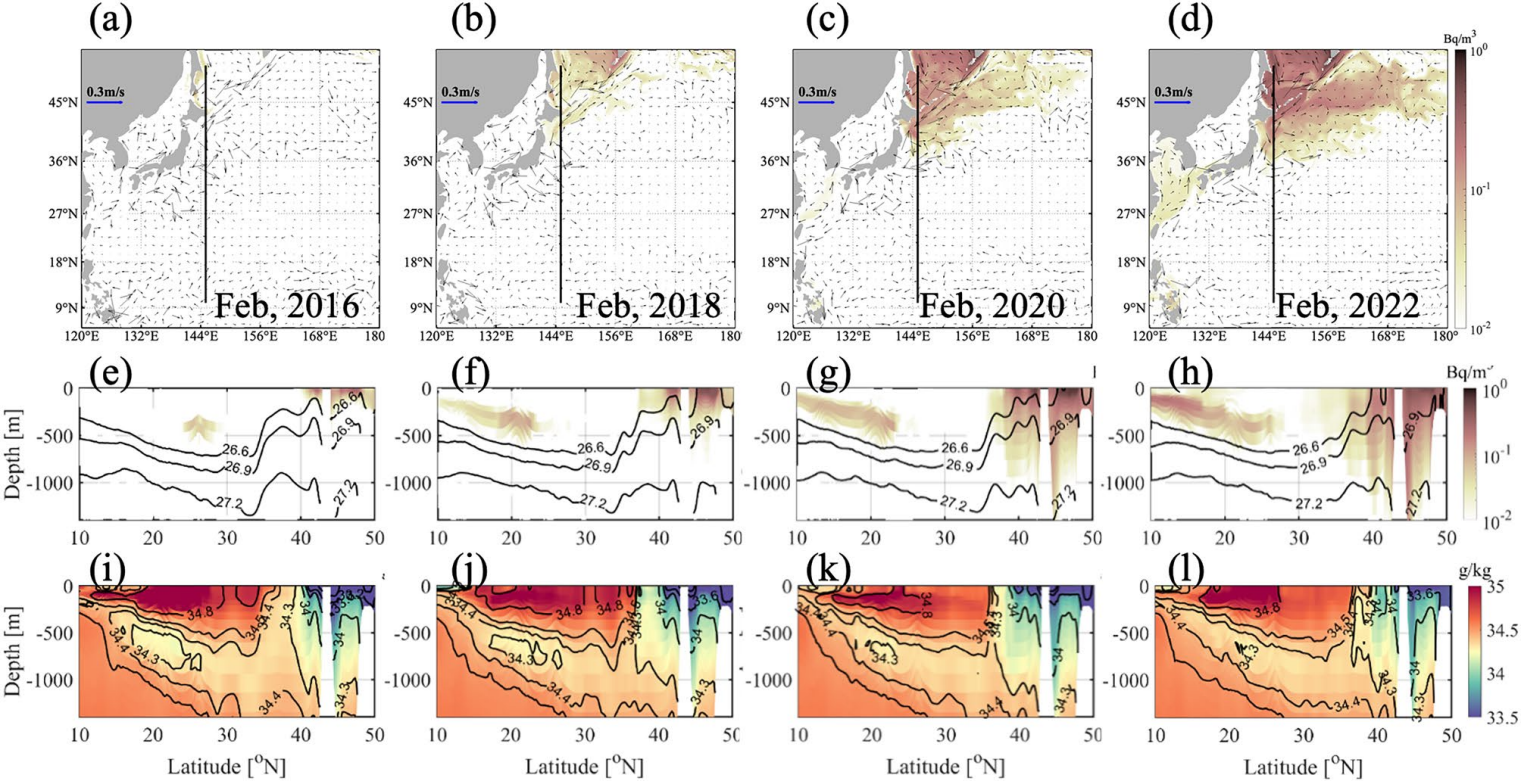
Monthly mean horizontal distribution of ^137^Cs concentration and surface current based on the model results at the density layer of 26.9 kg/m^3^, corresponding to North Pacific Intermediate Water (NPIW) density, in February (**a**) 2016, (**b**) 2018, (**c**) 2020, and (**d**) 2022. (**e**–**h**) Monthly vertical section of ^137^Cs concentration along 145°E during the same period. Background contour represents the potential density (kg/m^3^) corresponding to NPIW. (**i**–**l**) Monthly vertical section of salinity along 145°E during the same period. Figures were generated by S-TLee using MATLAB R2020a (http://www.mathworks.com).

The OIW formed in the Sea of Okhotsk moves south along the shoreline and crosses the SAF offshore^14^. Observations revealed radioactive material originating from the FDNPP in the southeastern part of Hokkaido around ten years following the accident, transported by the subarctic counterclockwise current^13^. Figure 4a–d show the horizontal concentration distribution of ^137^Cs at a density of 26.9 kg/m^3^ corresponding to the mid-depths of the OIW and NPIW every other February from 2017 to 2022. The increased ^137^Cs in the Sea of Okhotsk moved southward to the Kuroshio extension area along the coast. Figure 4e–h shows the vertical distribution of ^137^Cs at 145°E during this period. There were two cores of high concentration: one at a depth of 200–400 m south of 25°N and the other from the surface to a depth of approximately 1,000 m north of 35°N. The southern core may represent the dispersal of ^137^Cs through subtropical mode water^8^. Northern ^137^Cs was subducted from the surface at 50°N and moved southward along the layer at a density of 26.6–27.2 kg/m^3^. This density layer is consistent with the salinity minimum layer characteristic of NPIW (Fig. 4i–l). The vertical distribution pattern of ^137^Cs at approximately 40–50°N is similar to that of the minimum salinity layer. This result indicates that tritium, which is expected to be released in the near future, will increase in the Sea of Okhotsk at six years following release and move southward via the OIW. Thus, it may also distribute to the NPIW of the subtropical gyre.

As shown in Fig. 4c, ^137^Cs moved southward in 2020 and turned northeast via the OIW and NPIW in 2022. Radioactive materials delivered by the OIW are expected to follow two paths after 2022^15^: one moving southward to the Kuroshio extension region and entering the subtropical region, and the other moving eastward along the Kuroshio Extension Front and entering the subtropical region from the east of the North Pacific. Two paths were simulated from 2021 to 2022: a path gradually descending south along the NPIW density layer and an eastward path along the SAF (Fig. 4e–l).

### Atmospheric deposition effect of radioactive material

We conducted additional experiments on the atmospheric deposition of ^137^Cs to estimate its effects on the distribution of the radioactive concentration. The experiment with the atmospheric deposition will be referred to as the 'Atmosphere case' and considering only the oceanic release will be referred to as the 'Ocean case' henceforth. Supplementary Fig. S1 illustrates the surface distribution of monthly radioactive concentrations in the Atmosphere case. The total amount of atmospheric deposition of ^137^Cs was approximately 2.1 times that of the oceanic release, resulting in higher concentrations and a wider distribution in the surface layer of the Atmosphere case compared to that of the Ocean case. Results for the Atmosphere case show that radioactive materials were distributed in the Okhotsk Sea immediately after the accident and gradually decreased over time (Supplementary Fig. S1), whereas in the Ocean case, ^137^Cs reached the Okhotsk Sea from 2017 onwards, six years after the accident (Fig. 3f).

Supplementary Fig. S2 presents the ^134^Cs profiles of the observation^13^ and the model results. The profile locations for each year are indicated in Supplementary Fig. S2a. In the Ocean case, radioactive concentrations were remarkably increased six years after the accident. However, in the Atmosphere case, the initial increase in the concentration decreased over time and was lower than that in the Ocean case. Furthermore, the total radioactive concentration in the Atmosphere and Ocean cases were closer to the observed radioactive concentration in terms of the absolute values of the ^134^Cs profiles.

## Conclusions

We performed a tracer experiment to investigate the paths and travel times of radioactive material released from FDNPP in the subarctic region using a three-dimensional numerical model. The model simulated a marked increase in the amount of radioactive material observed in the Sea of Okhotsk over time. The travel time of radioactive material from the FDNPP to the Sea of Okhotsk via surface currents of the subarctic gyre was approximately six years. The results of both observation and the simulation model of the Sea of Okhotsk reveal that the maximum concentration of radioactive material was reached in the subsurface corresponding to the OIW layer. The radioactive material moved southward beneath the Kuroshio extension area via the OIW.

**Figure 5:**
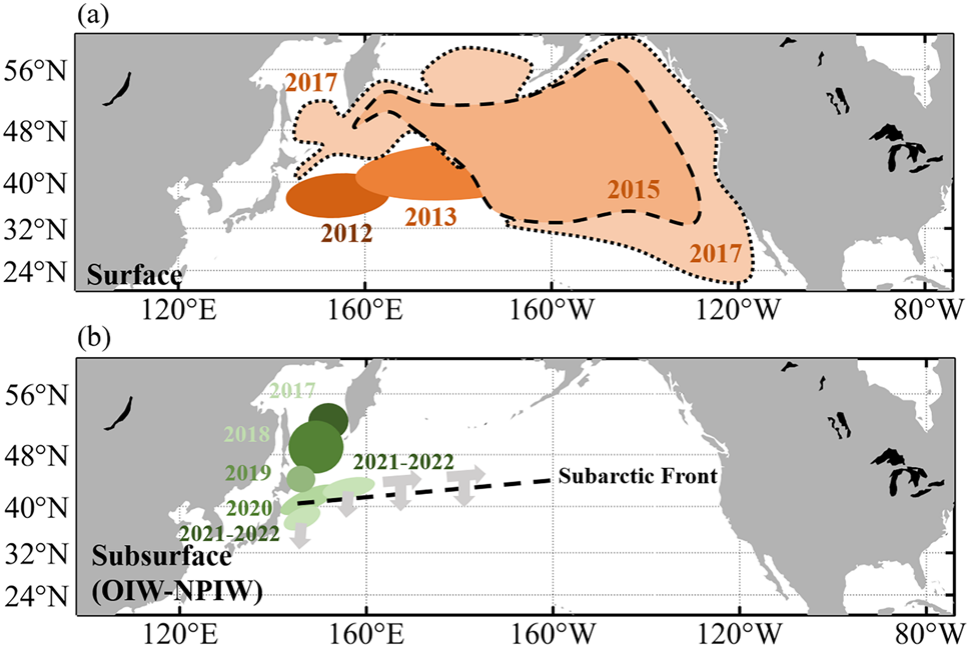
Schematic diagram of ^137^Cs paths induced by (**a**) surface current and (**b**) the Okhotsk Intermediate Water (OIW) and North Pacific Intermediate Water (NPIW) in the subarctic gyre region based on Figs. 3 and 4. The area and color of the ellipse roughly represent the distribution of ^137^Cs and year, respectively. Gray arrows represent the possible paths of ^137^Cs induced by the NPIW after 2022. Figures were generated by S-TLee using MATLAB R2020a (http://www.mathworks.com).

Figure 5 shows a schematic diagram of the path of radioactive material released directly into the ocean and moving by surface currents in the OIW and NPIW because of the FDNPP accident. The radioactive material moved mainly northeast owing to surface currents and was distributed widely in the subarctic region. The ^137^Cs that arrived in the Sea of Okhotsk by surface currents six years after the accident subsequently moved southward by the OIW in the subsurface to the Kuroshio extension area and turned northeast in 2020. Radioactive material dispersed eastward by the OIW has two paths: one leading directly to the subtropical region over the Subartic Front (SAF), and the other leading east along the SAF and entering the subtropical region east of the North Pacific Ocean.

The results of this study suggest that recirculation of radioactive material from the FDNPP may occur in the subsurface via the OIW and NPIW. The model simulated a marked increase in the amount of radioactive material observed in the Sea of Okhotsk over time, although there was some underestimation of the radioactive material concentration when only ocean release was considered. Our study has important implications for predicting the path and travel time of tritium planned for release at the FDNPP in the near future.

## Methods

### Definition of OIW and NPIW

Subduction of surface water occurs in the Sea of Okhotsk because of the increased density caused by surface cooling during cold winters^14–16^. The homogenous, oxygen-rich, OIW^14–16^ moves south along the coast and contributes to formation of the NPIW near the SAF. The NPIW is characterized by salinity and a potential vorticity minimum layer in the subtropical gyre. Its density and depth are 26.6–27.2 kg/m^3^ and 300–800 m, respectively^15,21–23^. In this study, the core densities of the NPIW and OIW were defined as 26.9 kg/m^3^.

### Model description

The Regional Ocean Modeling System, which has been widely utilized in numerous applications in oceanography, was used^24–27^. This modeling system is a free-surface, terrain-following ocean model that incorporates primitive equations. Using extended terrain-following coordinates, the basic equations were vertically discretized over the topography^28^, and then calculated on a staggered Arakawa C-grid in the horizontal plane.

The model domain used was the North Pacific region (Fig. 1, 98°E–76°W, 20°S–65°N). The model resolution of the horizontal spatial grid was 0.25°, with a time step of 300 s. The model domain had 30 vertical layers with sigma coordinates. A global relief model of the Earth’s surface with a spatial resolution of 1° was used for topography in this model^29^.

K-profile nonlocal closure schemes were utilized to parameterize vertical mixing^30^. The K-profile scheme was expanded to include surface and bottom oceanic boundary layers. Atmospheric forcing parameters were utilized using daily averages from the European Center for Medium-Range Weather Forecasts Reanalysis v5 (ERA5) datasets^31^. The following variables were included in atmospheric forcing: 2 m dew point temperature, mean sea level pressure, relative humidity, net short-wave downward radiance, 2 m air temperature, evaporation, precipitation, and 10 m wind velocities. Open boundary data, including temperature, salinity, and velocity, were obtained from Simple Ocean Data Assimilation version 3.4.1^32^.

The initial data were obtained from the World Ocean Atlas 2013 version 2, which consists of objective analyses and statistical data. The model was initialized with five years of spin-up with the forcing set to 2001 and was subsequently integrated for an additional ten years from January 2001 to March 2011. The resulting data from March 11, 2011, were employed as initial data for the tracer experiments. The initial data for the tracer experiments conducted in the present study were based on a previously published model^8^.

### Tracer experiment

We conducted a passive tracing experiment to trace ^137^Cs released from FDNPP. The governing equation of the passive tracers is expressed as follows (1).

1 $$\frac{\partial C}{{\partial t}} + \vec{v} \cdot \nabla C = - \frac{\partial }{\partial z}\left( {\overline{{C^{\prime } w^{\prime } }} - \nu_{\theta } \frac{\partial C}{{\partial z}}} \right) + {\mathbf{\mathcal{F}}}_{C} + D_{C}$$

where C is the concentration of the passive tracer,$\overrightarrow{v}$ is the vector velocity, $\overline{{C }^{\prime}{w}^{\prime}}$ is the turbulent tracer fluxes, ${\nu }_{\theta }$ is the molecular diffusivity, ${\mathcal{F}}_{C}$ is a forcing term, and ${D}_{C}$ is the horizontal diffusive term. The terms involving ${\nu }_{\theta }$ were disregarded because molecular viscosity-induced vertical mixing is less prevalent than turbulent mixing. The vertical diffusivity coefficients estimated from the K-profile nonlocal closure schemes were used to determine the turbulent tracer fluxes. The horizontal diffusivity coefficient was set to 20 m^2^/s. No sinks or sources were assumed during the experiments. Therefore, the term ${\mathcal{F}}_{C}$ was disregarded. The initial value of the tracer was taken to be 3.5 PBq^33^.

The passive tracer was released at a surface grid (141.25°E and 37.38°N) near the FDNPP on March 11, 2011. The released passive tracer was dispersed via advection and diffusion and then tracked for approximately 12 years from March 11, 2011, to December 31, 2022. Note that this extends the simulations reported in previous study^8^ by an additional two years. The boundary data from 2021 to 2022 were used as boundary values for 2020 because Simple Ocean Data Assimilation data are available only until 2020. To consider the half-life of ^137^Cs (30 years), the concentration of ^137^Cs was calculated by applying a half-life function of 30 years at each time step.

### Model validation

The model successfully simulated the distribution of radioactive material at the surface and subsurface of the North Pacific Ocean^8^. The model accurately simulated the sea surface temperature (SST) in the Okhotsk Sea, as compared with satellite-observed SST. Supplementary Fig. S3 displays the monthly mean SST of the Operational Sea Surface Temperature and Ice Analysis and model results for the Okhotsk Sea in February and August 2012, 2014, and 2016. The model well-simulated the Kuroshio Front and SST of the Sea of Okhotsk during the summer and winter seasons, as shown in the observations.

To evaluate the model performance in terms of simulating the NPIW, which is characterized by a minimum salinity layer, we compared a vertical section of temperature, salinity, and potential density from the Sea of Okhotsk to the Kuroshio extension (Supplementary Fig. S4a) with the values from the observed (EN4) data. The model simulated a temperature similar to that of EN4, with a vertical gradient that decreased northward (Supplementary Fig. S4b,e). The model well-simulated the salinity minimum layer (Supplementary Fig. S4c,f). Low salinity up to a depth of approximately 400 m at 40–50°N latitude was well-simulated, and a minimum salinity layer extending to a depth of 400–800 m at 15–40°N latitude was well-represented in the model (Supplementary Fig. S4d,g). The model well-simulated the observed depth and thickness of the NPIW, with density of 26.7–26.9 kg/m^3^. The comparable results between EN4 and the model support that the model accurately simulates the NPIW.

### Atmospheric deposition experiment

The passive tracer corresponding to the atmospheric deposition of ^137^Cs was released at the surface layer as a surface boundary from March 11 to April 30 in 2011. There is large uncertainty in the amount of atmospheric deposition of ^137^Cs, ranging from 5 to 15 PBq^1,34–37^. Considering the median value reported in previous studies, the total amount of atmospheric deposition of ^137^Cs was set to 7.5 PBq. The spatial distribution of ^137^Cs was reproduced from a previous study^1^ (Supplementary Fig. S5). From March 11 to April 30, the same amount of ^137^Cs was set to enter into the surface over time with the same spatial distribution, although it shows temporal changes in the previous study^1^. Other features of the experiment on atmospheric deposition were consistent with the ocean tracer experiment.

### Supplementary Information

Supplementary Figures.

## Data Availability

The data supporting the conclusions of this study are available from the corresponding author upon a reasonable request.
