# Supplementary material for: Surface and subsurface dispersal of radioactive materials from Fukushima by subpolar gyre and intermediate waters in the North Pacific
Source: Sci Rep. 2024 Mar 1;14:5055. doi: 10.1038/s41598-024-55328-7 (PMC10904853; doi:10.1038/s41598-024-55328-7)
Supplement: Supplementary file 1 — Supplementary Figures. [file 41598_2024_55328_MOESM1_ESM.docx]

**Surface and subsurface dispersal of radioactive materials from Fukushima by subpolar gyre and intermediate waters in the North Pacific**

Seung-Tae Lee, Yang-Ki Cho, Jihun Jung, Seunghwa Chae

**SUPPLEMENTARY FIGURES**

**
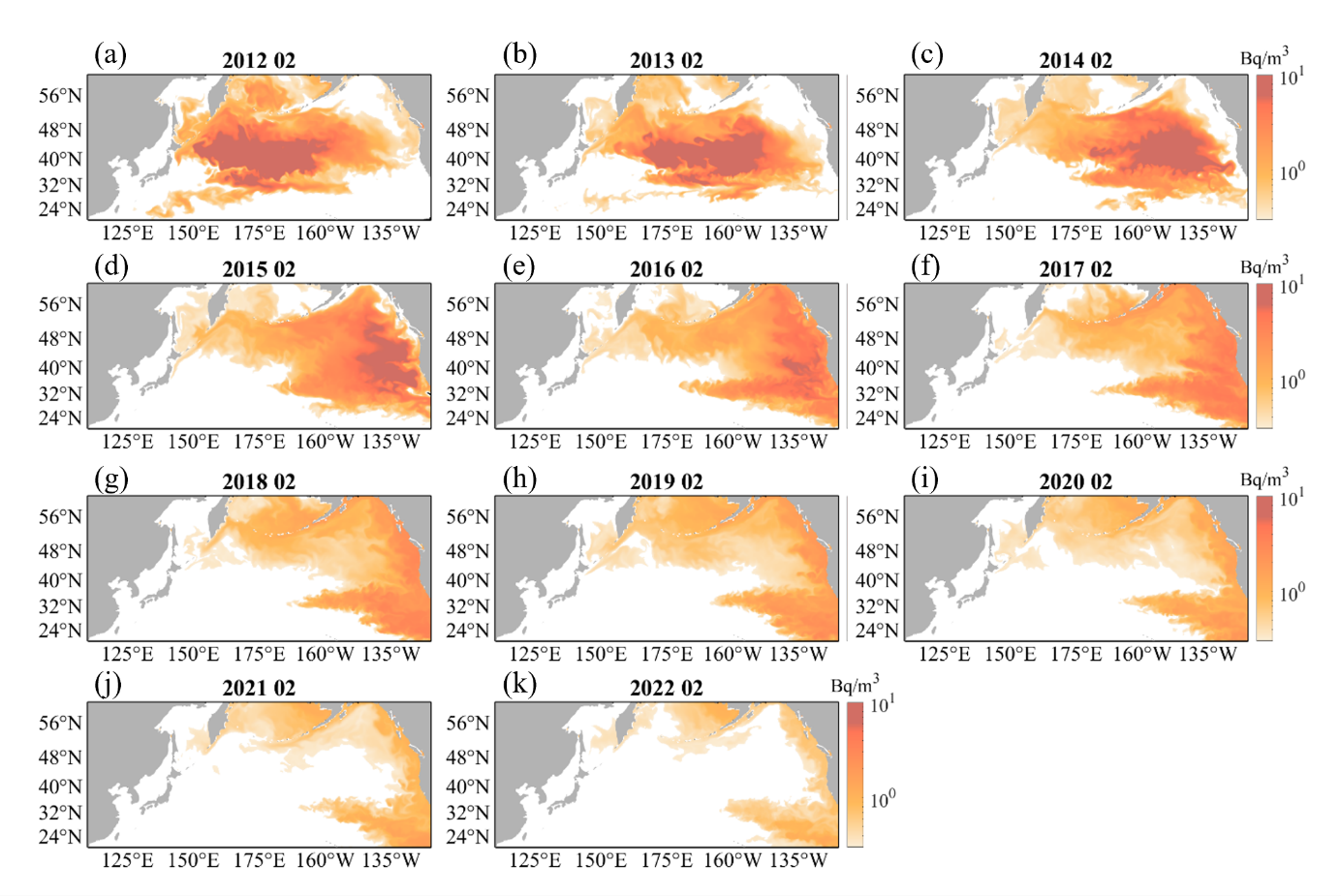
**

**Figure. S1.** Monthly surface distribution of ^137^Cs concentration measured every February from (a–k) 2012 to 2022, based on the result of an atmospheric deposition experiment. Figures were generated by S-T Lee using MATLAB R2020a (http://www.mathworks.com).

**
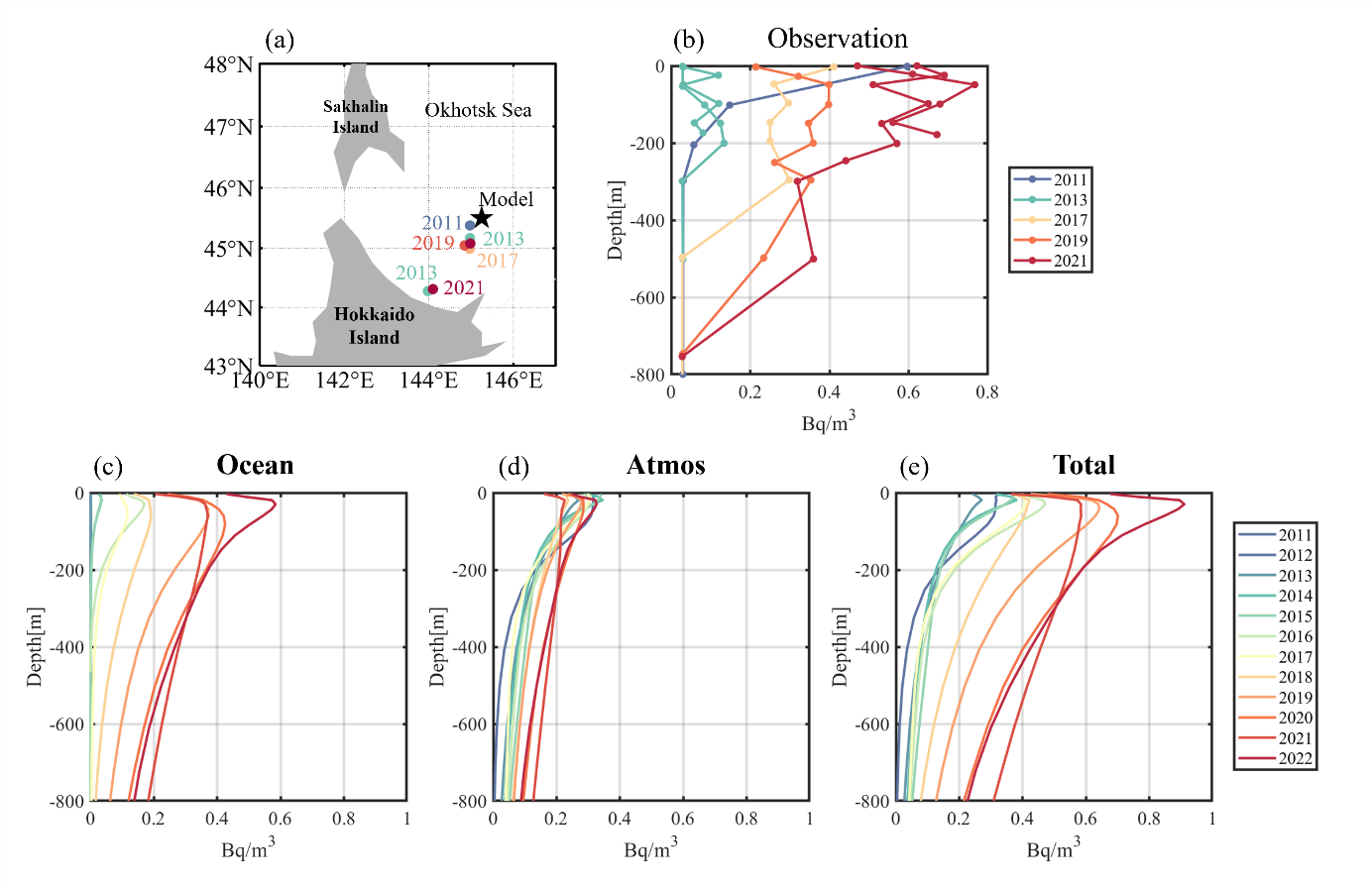
**

**Figure. S2.** Comparison of observations and model simulation data in the southern area of the Sea of Okhotsk. (a) Observation stations utilized by Inoue et al [14] and the selected model point in the current study. (b) ^134^Cs concentrations decay-corrected to the date of the FDNPP accident in July, from Inoue et al [14]. Simulated ^134^Cs concentrations in July without the half-life decay of ^134^Cs from (c) Ocean, (d) Atmosphere and (e) Total cases. Figures were generated by S-T Lee using MATLAB R2020a (http://www.mathworks.com).

**
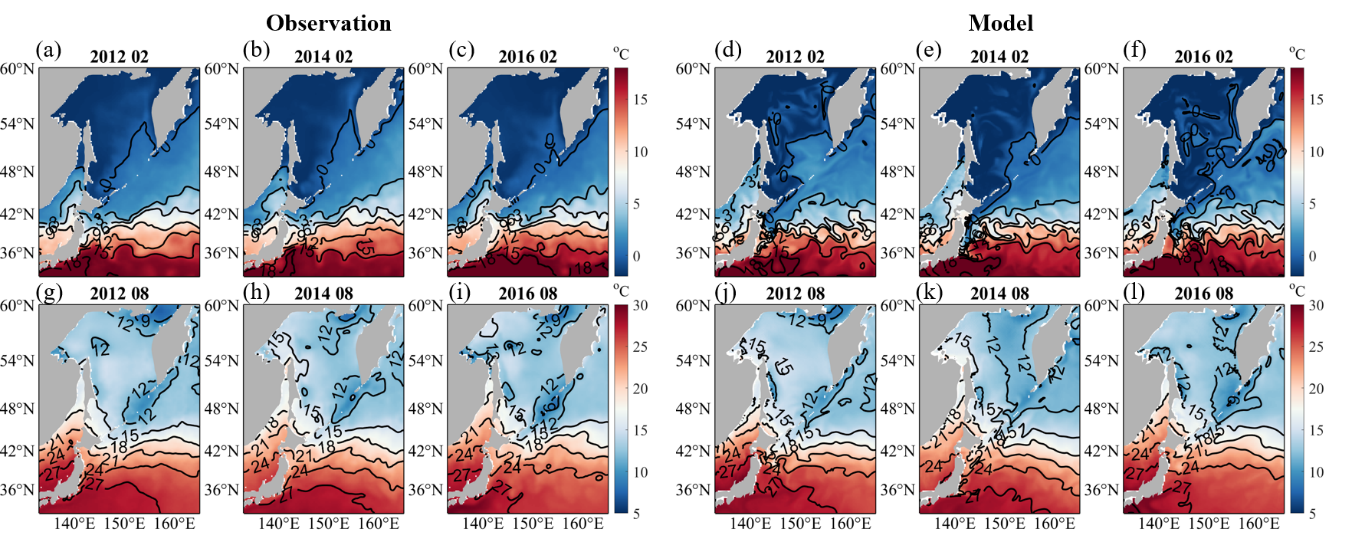
**

**Figure. S3.** Comparison of the monthly mean sea-surface temperature between the (a–c, g–i) OSTIA and (d–f, j–l) model results for the Okhotsk Sea in February and August 2012 (a,g,d,j), 2014 (b,h,e,k), and 2016 (c,i,f,l).

**
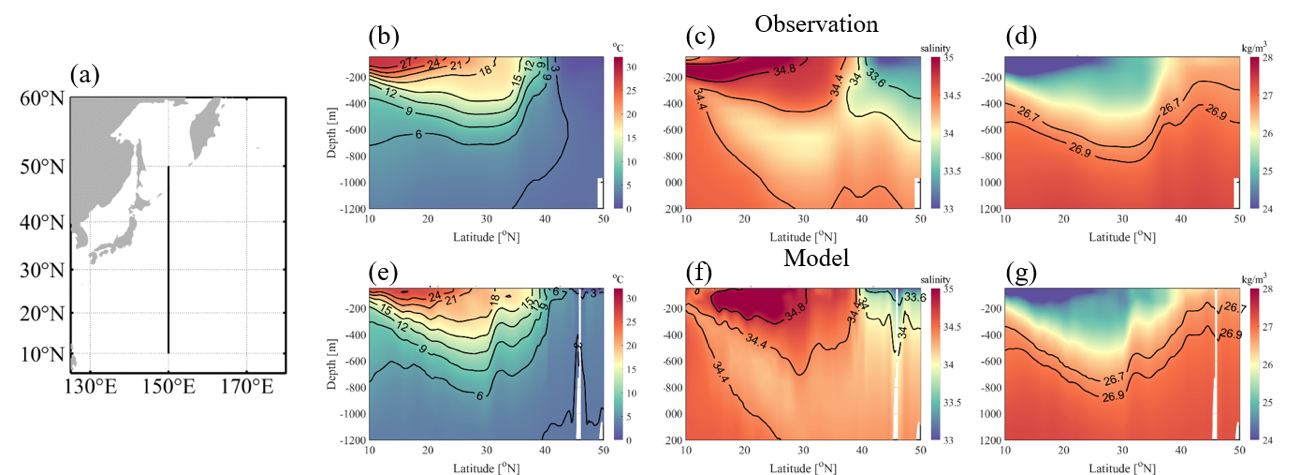
**

**Figure. S4.** (a) Selected line for the comparison of vertical sections between EN4 and the model. Vertical sections of (b and e) temperature, (c and f) salinity, and (d and g) potential density from (b–d) EN4 and (e–g) the model. The background contour lines of the potential density represent the potential density layer corresponding to the NPIW.

**
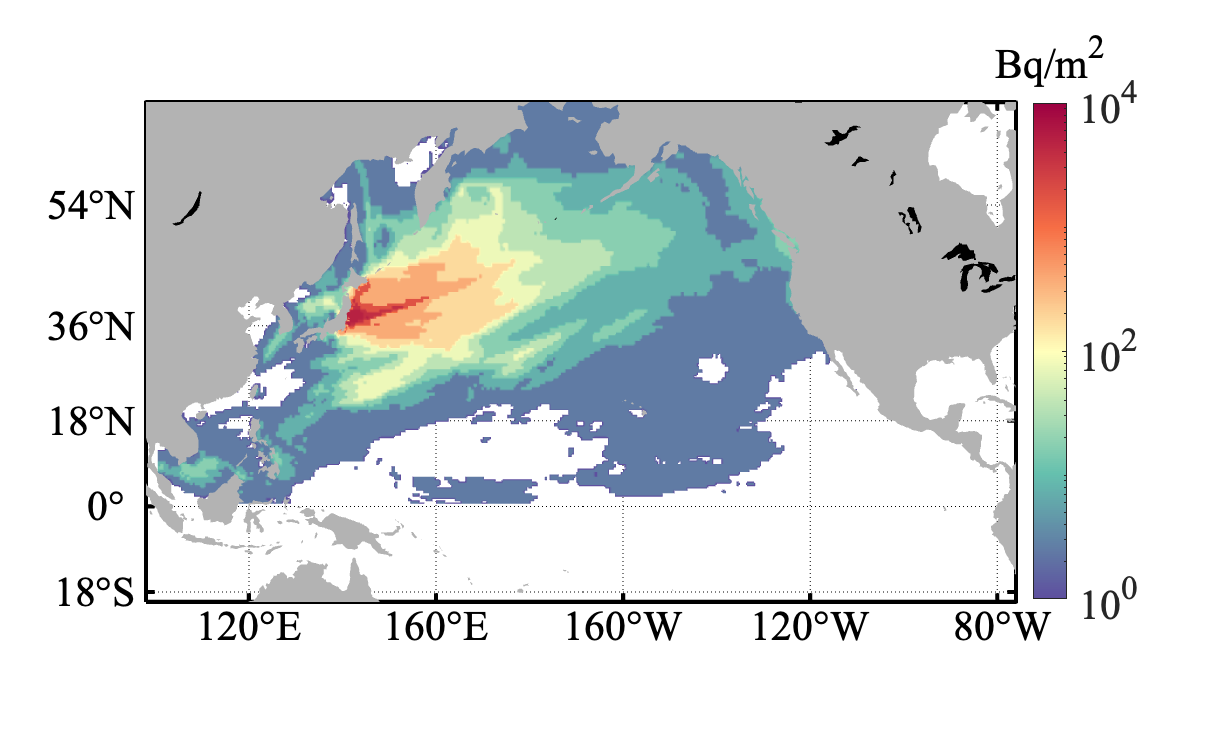
**

**Figure. S5.** Total input ^137^Cs amount for the atmospheric deposition in the Atmosphere case from March 11 to April 30. The distribution of atmospheric deposition was referenced from Aoyama et al. (2016)
